# Management of a Recurrent Lower Lip Mucocele in a Pediatric Patient: A Case Report

**DOI:** 10.7759/cureus.98166

**Published:** 2025-11-30

**Authors:** Mahdi Ayoub, Elias Ghosein

**Affiliations:** 1 Faculty of Dentistry, Lebanese University, Beirut, LBN

**Keywords:** mucocele, oral pathology, oral surgery, pediatric, surgical excision

## Abstract

Mucoceles are common, benign cystic lesions of the minor salivary glands, most often found on the lower lip of young patients. While they are usually diagnosed clinically, histopathological examination is essential for confirmation and to rule out other lesions. The primary etiology is trauma to the salivary gland duct, which leads to the extravasation of mucus and the formation of a pseudocyst.

This case report details the successful surgical excision of a recurrent lower lip mucocele in a 13-year-old female patient. The patient had a history of recurrence following a prior laser surgery. A standard scalpel excision was performed, and histopathological analysis confirmed a mucus extravasation mucocele. Follow-up visits at one month and six months revealed complete healing with no signs of recurrence, demonstrating that meticulous surgical excision remains a definitive and effective treatment for pediatric oral mucoceles.

In our case, meticulous surgical excision was an effective treatment for a pediatric oral mucocele, ensuring complete resolution and preventing recurrence.

## Introduction

Mucoceles represent a common benign cystic pathology that arises from the minor salivary glands located within the oral mucosa [1,2]. These lesions are characterized as cavities containing an accumulation of mucus [1,3]. The lower lip is the most frequent site for these lesions, constituting 80% of all cases, and their etiology is commonly linked to chronic trauma or parafunctional habits such as lip biting or sucking, aggravated usually by the presence of an orthodontic treatment [4].

The general incidence of mucoceles is approximately 2.5 lesions per 1000 patients [3,5]. They occur across all age groups but show a peak incidence in the second and third decades of life, specifically between 10 and 29 years, affecting both genders [2-6]. In some studies, mucoceles comprise a significant percentage of oral biopsies, such as 5.6% of all oral biopsies examined [6]. In a separate study focusing on lip lesions, mucoceles accounted for 32.9% of all lip lesions examined in one institution [7].

Mucoceles develop via two distinct pathogenic mechanisms: extravasation and retention [1,3]. The extravasation type results from the rupture of a salivary gland duct or acini, often initiated by physical trauma or habits such as lip-sucking or biting, which causes the leakage of fluid into the surrounding submucosal tissue [1-4]. This accumulation of stagnant mucus forms a cyst-like space that is characteristically encapsulated by granulation tissue rather than a true epithelial lining [4-6].

Conversely, retention mucoceles arise from the obstruction of a salivary gland duct, which impedes glandular secretion, leading to ductal dilation and subsequent surface swelling [1]. This latter type is a true cyst, distinguished by a cavity lined with a clearly defined cuboidal epithelium, and typically presents with a lower degree of inflammation [3,4,6]. The extravasation-type mucocele is the predominant form, accounting for 84.48% of cases, as opposed to the less common retention type, which constitutes 15.52% [2,3].

The diagnosis of a mucocele relies on four key clinical factors. Its frequent lower lip location is critical due to the high incidence of trauma in this area. A history of trauma is essential, as this mechanism causes the underlying salivary duct rupture and mucin extravasation. The rapid onset helps differentiate it from true neoplasms, and the observation of variations in size and consistency (swelling and shrinking) provides strong evidence for this dynamic lesion [8].

The standard therapeutic approach is conservative surgical excision with a scalpel, though alternative modalities such as electrosurgery, cryosurgery, micro-marsupialization, marsupialization, intralesional steroid injections, and laser therapy (e.g., diode laser) are also employed. To minimize the risk of recurrence, it is imperative that the excisional procedure includes the removal of any associated minor salivary glands contributing to the lesion [1,5].

Recurrence rates are variable, generally reported between 10% and 20%, but can reach as high as 40%. Notably, a systematic review and meta-analysis by Hashemi et al. indicates no statistically significant difference in recurrence rates among surgical excision, CO_2_ laser therapy, and marsupialization techniques [9].

This case report describes the surgical excision of a lower lip mucocele in a pediatric patient, highlighting the importance of determining the treatment option in recurrent mucoceles cases management and providing a practical example for clinicians.

## Case presentation

**Figure 1:**
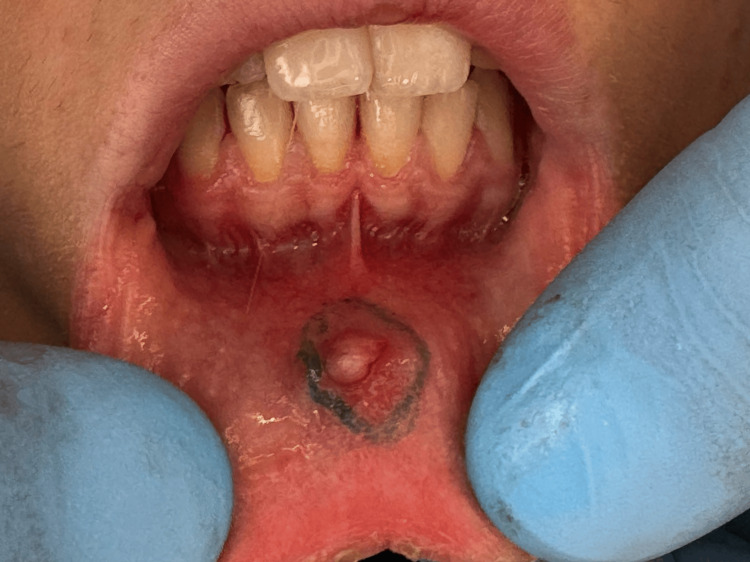
Clinical photograph showing a mucocele located on the inner aspect of the lower lip. Dimensions: 0.5x0.4 cm. The preoperative marking is the incision line.

A 13-year-old female patient presented with a soft, painless, and gradually enlarging localized swelling on the inner surface of her lower lip along the midline, a condition present for four months prior to the visit (Figure 1), clinically consistent with a mucocele. The patient's medical history was non-contributory and the patient's parents did not report any habit or trauma. The patient had undergone a laser surgery for the same lesion eight months prior to presenting at our clinic; however, the lesion recurred.

An oral and maxillofacial surgeon performed a complete surgical excision of the lesion, and the excised specimen measured 0.5x0.4cm. The primary closure was achieved using a 4-0 polyglycolic acid suture and compression for 10 minutes was enough for complete hemostasis. The patient was prescribed analgesics post-procedure.

**Figure 2:**
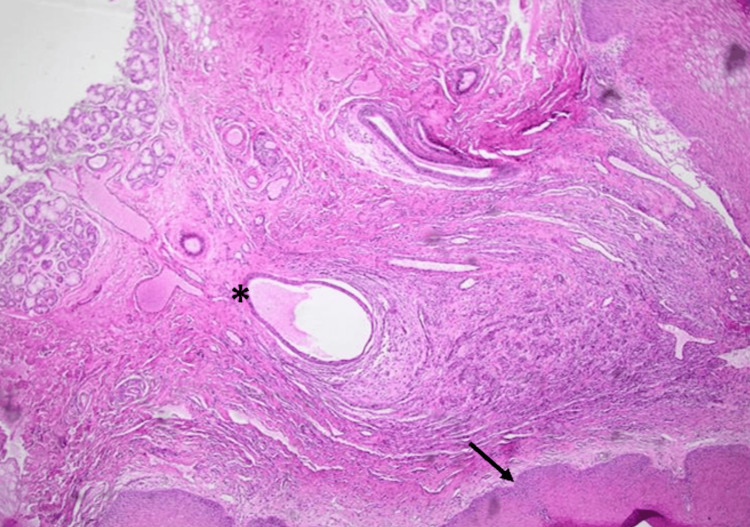
Mucocele (H&E, x10): Large mucin pool within a cystic space (asterisk) surrounded by a connective tissue stroma including fibroblasts. The epidermis seen below is acanthotic (black arrow).

**Figure 3:**
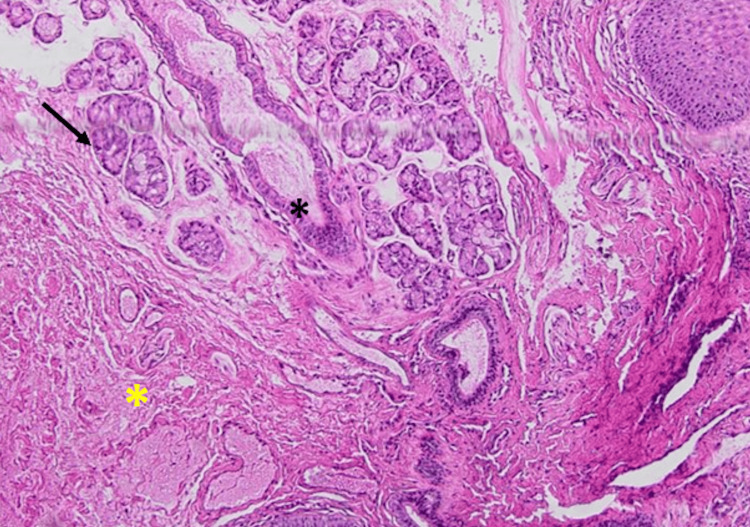
Mucocele (H&E, x10): Dilated mucin pools (black asterisk) in connective tissue stroma surrounded by granulation tissue, inflammatory infiltrate, and mucinophages (yellow asterisk). There is no true epithelial lining; adjacent minor salivary glands are seen (black arrow).

The excised specimen was preserved in 10% formalin and submitted for histopathological examination. The pathology report confirmed the diagnosis of a salivary mucocele, describing a superficial cystic cavity devoid of an epithelial lining, surrounded by a fibrous wall with chronic inflammatory infiltrate as shown in Figure 2, and an associated minor salivary gland. No evidence of malignancy was reported. Figure 3 shows the salivary gland tissue.

**Figure 4:**
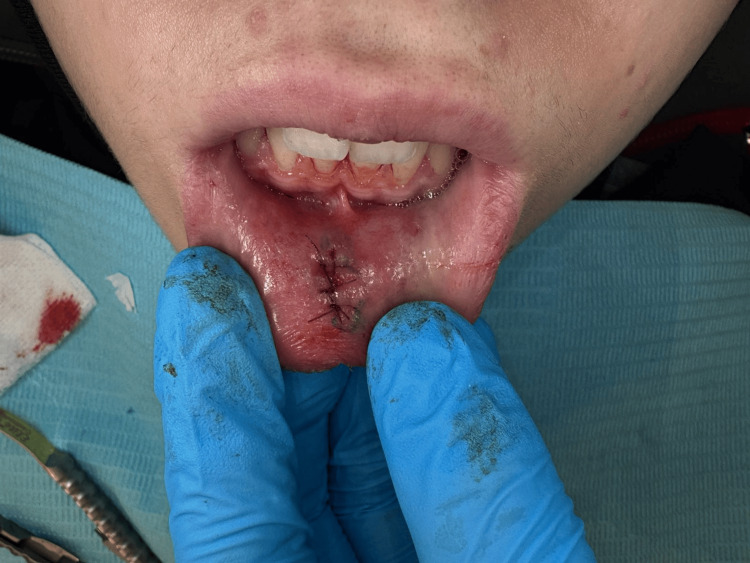
Clinical image showing the primary closure assured by sutures post-operatively.

Based on the clinical presentation of a localized labial swelling, the clinical differential diagnosis should include other entities such as fibromas, lipomas, hemangiomas, salivary duct cysts, and even malignant neoplasms like mucoepidermoid carcinoma. In the present case, however, no significant diagnostic challenges were reported, as the clinical features were strongly suggestive of a mucocele [10]. At the one-week postoperative follow-up, sutures were removed, revealing a clean, completely closed surgical site with satisfactory healing (Figure 4), indicating a resolution of this common benign lesion.

**Figure 5:**
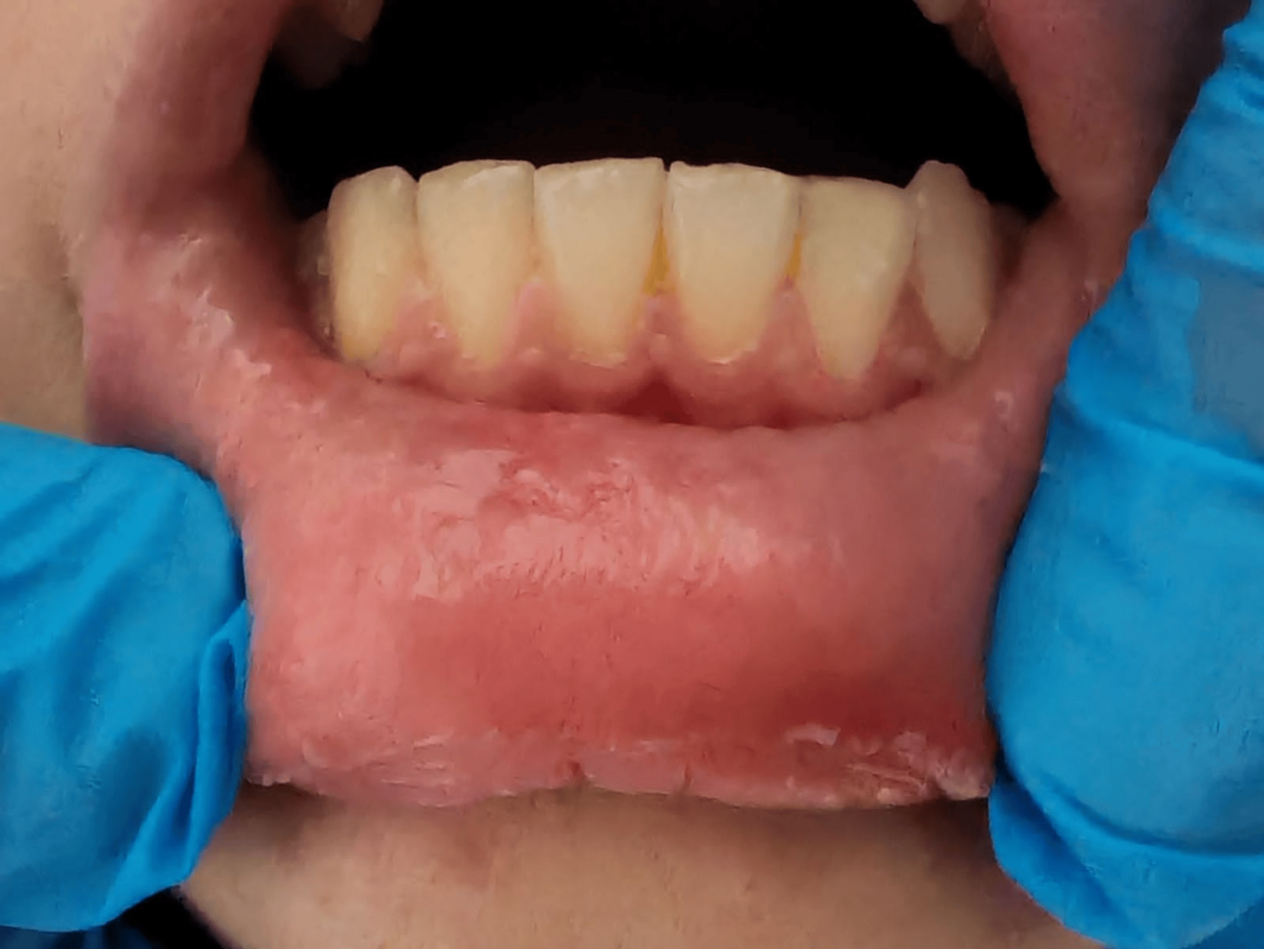
Clinical image at the one-month follow-up showing the surgical site on the lower lip with complete healing and no evidence of recurrence.

At the one-month postoperative follow-up, the site shows good healing and no signs of recurrence (Figure 5).

**Figure 6:**
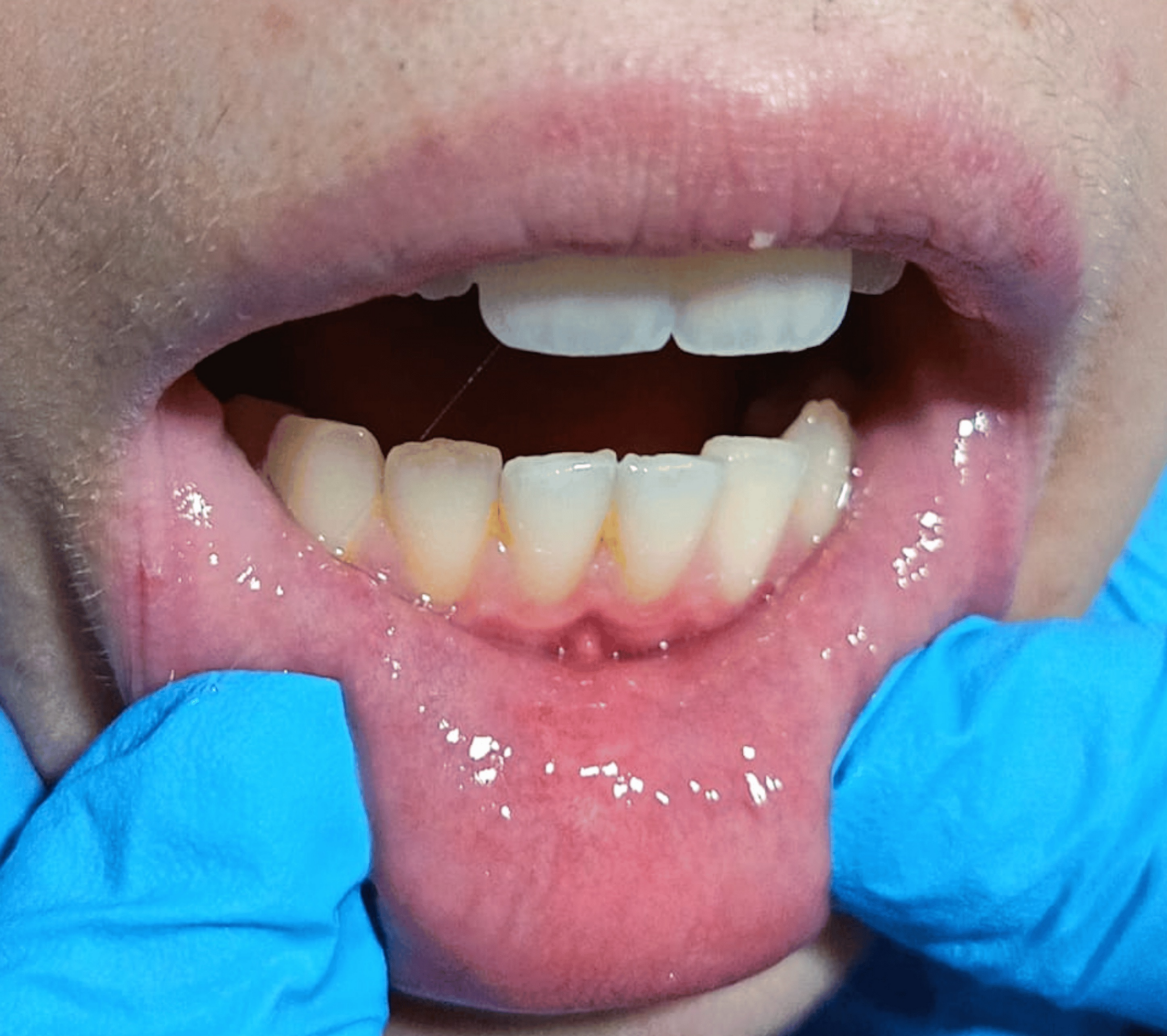
Six-month follow-up evaluation of the lower lip, confirming the absence of disease recurrence

The patient and her parents expressed satisfaction with the treatment outcomes, noting that the procedure was painless and there was no pain or discomfort during or after the intervention. Figure 6 illustrates the findings at the six-month follow-up evaluation; clinical examination confirmed the absence of disease recurrence, and the patient reported a high level of satisfaction with the therapeutic outcome.

## Discussion

This case report details the successful surgical excision of a lower lip mucocele in a 13-year-old female patient. The patient initially presented with a localized, soft, and painless swelling on the lower lip that had gradually increased in size for the past four months prior to the initial visit, a presentation consistent with the classic clinical features of a mucocele [1,6,9].

Definitive diagnosis was established through histopathological examination following surgical removal. The histopathological analysis revealed a small, superficial cystic cavity lacking an epithelial lining, surrounded by a fibrous wall expanded by chronic inflammatory infiltrate and spilled mucin. These findings confirmed the diagnosis as a mucus extravasation phenomenon, which constitutes a pseudocyst formed by the rupture of a salivary gland duct and the subsequent leakage of mucus into the surrounding soft tissue [2-6].

Recurrence of this type of lesion typically manifests within the initial months following surgical intervention [1-4]. No signs of recurrence were observed during the follow-up period of six months (Figure 6), indicating successful treatment.

The clinical characteristics observed in this case align well with established epidemiological data on oral mucoceles. Mucoceles are common oral mucosal lesions [2,4], with a peak incidence noted in the second and third decade of life (10-29 years), and this patient, at 13 years old, falls within this high-incidence demographic [2,4-7].

Furthermore, the lower lip consistently represents the predominant anatomical site, accounting for 44% to 81.9% of the reported cases across various studies. This patient's presentation aligns entirely with established patterns, though the underlying reasons for this anatomical predilection remain unclear [1,2,4-6].

The pathogenesis in this case is strongly suggestive of a trauma-induced mechanism, which leads to the rupture of a minor salivary gland duct and subsequent extravasation of mucin. This process results in the formation of a pseudocyst, a mechanism confirmed by the histopathological finding of a mucocele lacking an epithelial lining, which is characteristic of the extravasation type [1,4].

Regarding management, conventional surgical excision is considered the standard treatment for mucoceles [10,11]. This approach aims for the complete removal of the lesion along with any associated salivary gland tissue to minimize the risk of recurrence [1,2,5,10].

Although recurrence rates for mucoceles can vary, typically ranging from 10% to 20% and occasionally reaching as high as 40% in some studies [2,9], the successful resolution in this case, with no recurrence observed at six months follow-up, underscores the effectiveness of this standard surgical intervention when meticulously performed.

For larger lesions, marsupialization presents a viable alternative, while more contemporary techniques such as laser ablation and micro-marsupialization have gained popularity [3,5,9,11]. These modalities are particularly advantageous in the pediatric population, offering benefits such as reduced postoperative pain, minimal intraoperative bleeding, shorter procedural times, and less scarring, which are valuable in managing a patient cohort often challenged by dental anxiety and the need to avoid general anesthesia [11].

The initial clinical diagnosis of a suspected mucocele was based on its presentation as a painless, soft, and fluctuant swelling of the lower lip [2,6,8]. However, definitive confirmation required histopathological examination, which is crucial for distinguishing mucoceles from other clinically similar oral lesions and excluding malignancy [3,5,8].

The differential diagnosis for such a presentation must include entities such as fibromas, which are firm nodules resulting from chronic irritation, and more critically, mucoepidermoid carcinoma, a malignant salivary gland tumor that can present as a swelling and may mimic mucoceles histologically if salivary acini are absent [2,8,10].

In this case, the histological findings of spilled mucin surrounded by granulation tissue and the distinct absence of an epithelial lining definitively confirmed the diagnosis as a mucus extravasation phenomenon [10]. This concurrently ruled out other conditions like true retention cysts, which are characterized by an epithelial lining, and other neoplastic processes, underscoring that accurate histopathological assessment is essential for a correct diagnosis and appropriate management [2].

Conventional surgical removal is the most common and effective treatment for mucoceles [12,13]. Using an elliptical incision is a popular technique because it minimizes the loss of mucosal tissue, reduces the risk of scarring, and prevents the contents of the cyst from spilling, which can help prevent recurrence [12].

A key aspect of successful management observed in this case was the positive patient-centered outcome. The patient's parents expressed satisfaction with the treatment results, while the child reported the procedure as painless, experiencing no discomfort during or after the intervention. The postoperative course was uneventful, with satisfactory wound healing and complete closure of the surgical site observed at the one-week review, showing no signs of infection or complications. This atraumatic recovery, coupled with adherence to follow-up visits, constitutes a crucial element contributing to favorable long-term outcomes and emphasizes the importance of comprehensive patient care, especially within the pediatric population.

## Conclusions

In summary, this case illustrates that meticulous surgical excision is a highly effective treatment modality for pediatric oral mucoceles. The successful outcome, characterized by complete healing and an absence of recurrence, underscores that favorable results are intrinsically linked to the precision of the surgical technique and the patient per-op and post-op cooperation. This approach remains a robust first-line option, capable of ensuring the comprehensive removal of the lesion and its associated glandular tissue.
